# P-1574. Impact on Infectious Diseases Division Revenue with Documentation and Billing Training Programs

**DOI:** 10.1093/ofid/ofaf695.1754

**Published:** 2026-01-11

**Authors:** John E McKinnon, Gina E Maki, Laura Sublett, Marcus Zervos, Cassandra Salgado

**Affiliations:** Division of Infectious Diseases, Department of Medicine, Medical University of South Carolina,, Charleston, SC; Henry Ford Health, Detroit, Michigan; Medical University of South Carolina, Charleston, South Carolina; Henry Ford Hospital, Detroit, Michigan; Medical University of South Carolina, Charleston, South Carolina

## Abstract

**Background:**

Improving revenue for Infectious Diseases (ID) divisions has gained importance in recent years as CMS reimbursement has continued to decline and rules have negatively impacted revenue overall. Improving documentation, collaborating with hospital billing services and addressing CMS rule changes can impart a significant impact on divisional revenue.

**Methods:**

We instituted an intensive intervention program within the ID division at Henry Ford Health (HFH) from 2017 to 2021, followed by a low intensity intervention program from 2022-2025. At the Medical University of South Carolina (MUSC),a low intensity program was initiated in 2023. These training and monitoring programs were performed for ID providers and fellows directing education, documentation, coding and billing services. We coordinated with institutional billing and coding services. Revenue value units (RVUs) are considered reimbursed at 2025 CMS rate.

**Results:**

From 2016 to 2021, HFH ID program reduced level 1 billing for new inpatients from 5% to 0.96%, level 2 from 33% to 11.55%, while increasing level 3 billing from 62% to 87.48%. Similarly, increased the proportion of level 3 billing for subsequent visits from 34% to 69.61%, with decreases in level 1 & 2 billing rates. RVUs gains from 2016 to 2021 were 704.19 per 1k patient contacts, representing an increase of $421,324 from improved billing documentation in 2021. From 2022 to 2025, level 1 initial and subsequent notes remained < 1%. 2023 CMS rule changes impacted Level 3 initial and subsequent note billing, down to 48% and 28.6% in 2023 recovering to 71.8% and 52.4% respectively, by 2025. At MUSC, from 2022 to 2025 the low intensity billing intervention achieved increasing inpatient level 3 billing for initial 16% to 25.5% and subsequent 24.84% to 38.27% notes. MUSC improvement in RVUs from 2022 to 2024 were 113.2 RVUs per 1k patient contacts, representing an increase of $32,153 for fiscal year 2024 in revenue.

**Conclusion:**

Both high and low intensity coding and billing programs directed by ID physicians can improve documentation, increase billing and divisional revenue, as well as address new coding rule changes. Higher intensity documentation programs with ID faculty support have the potential for further improvements in billing.

**Disclosures:**

John E. McKinnon, MD, MSc, FIDSA, EMD Serono: Advisor/Consultant Marcus Zervos, MD, merck: Honoraria

